# Philadelphia County Medical Society

**Published:** 1891-08

**Authors:** 


### PHILADELPHIA COUNTY MEDICAL SOCIETY.

*Stated Meeting, May 29, 1891.*

THE President, JOHN B. ROBERTS, M. D., in the Chair.

Dr. FRANK WOODBURY submitted a paper on

### NITROGEN–CONTAINING FOODS AND THEIR RELATIONS TO MORBID STATES.

In connection with the paper of the evening, by Prof. Chittenden, upon the Food-value of Beef-preparations, I have been invited by the Honorable Board of Directors to contribute a few remarks upon Nitrogen-containing Foods and their Relations to Certain Morbid States. Under the circumstances, it is proper that what I have to say shall be made as brief as possible.

At the outset, our attention is drawn to some fundamental physiological facts which must be kept in mind during the discussion of this subject. The human body is now regarded as a unit composed of an aggregation or community of cells. These anatomical elements differ from each other in some respects, but agree in this: each cell consists of two parts, one living and one non-living, corresponding with cell-nucleus and formed material. What is visible to us is the non-living part, or the formed material; the real living part of the organism is hidden from view. Just as in vegetable tissue, the parts that are permanent and solid are composed of the cell-walls, which may remain long after the essential living part or protoplasm of the wood-cell has dried up and disappeared—in a similar way, in the human subject, the various organs and tissues which give it form and substance are not living; the only part exhibiting vital phenomena is the soft, shapeless, and colorless cell-nucleus, consisting of protoplasm or bioplasm. This living substance, in its chemical composition, resembles the various tissues, varying somewhat according to function, but it contains one essential ingredient which is so characteristic as to confer its name upon the whole class—this element is nitrogen. The celebrated dictum, "Without phosphorus, no thought," might be paraphrased "Without nitrogen, no life." Viewed from the physiological standpoint, the name "Azote," applied to this element by Lavoisier, appears remarkably inappropriate.

As a necessary constituent of the tissues, therefore, nitrogen, in a state of combination, is always present in the human body. Since it is found in considerable quantity and in various forms in the excretions, some two or three hundred grains being discharged daily by the kidneys alone, besides what is lost by the intestinal tract and the skin, it is evident that in order to maintain life the supply must be kept up from outside sources. There are two principal directions in which we may look for the supply of nitrogen: (1) the atmospheric air, and (2) the food.

Although the atmospheric air contains about eighty per cent. of nitrogen, we may dismiss this at once as not available, beyond a very limited extent. Experiment has shown that it is not consumed or absorbed in the act of respiration; but a certain amount of air is always swallowed with the food and passes into the stomach, where it may become absorbed by the gastro-intestinal mucous membrane. It is possible that a small quantity is introduced by this channel, especially since it has been demonstrated that a moderate amount of gaseous nitrogen is excreted or exhaled by the skin.

Nitrogen-containing food must, therefore, be regarded as practically the only source of the constant supply of nitrogen which is so essential to the maintenance of the body in a normal condition. In fact, due attention has already been given to this by Liebig, Fick, Wislicenus, Parkes, Pavy, Flint, and others; and the proper relation of the two great divisions of proximate principles of organic origin, the nitrogenized and the non-nitrogenized, have been pretty closely determined. As their results are to be found in all the text-books, I will not refer to them in detail. I may remark, however, in passing, that, from the clinical standpoint, there appears to be a fallacy underlying all these calculations of dietaries, where food values are expressed in grains of nitrogen and carbon, inasmuch as no allowance is made for waste; the entire quantity ingested is supposed to be digested and assimilated. In practice, we know that the feces contain considerable nitrogen, which is not excretory, properly speaking, but represents the excess of consumption, part of the food having escaped digestion. In nursing infants the feces consist largely of undigested casein. Even adults are not able to entirely digest milk, and if so simple an article of food as milk is not completely assimilated, what warrant have we for assuming that the nitrogenized constituents of peas and beans, or of animal tissue, will yield their full equivalent of potential force to the organism? On the contrary, we know it to be a fact, that much food-stuff passes through the alimentary canal without having its proximate principles extracted by the digestive organs and the absorbents.

We may, however, both clinically and by physiological experiment, make due allowance for the personal equation, determine with sufficient exactness the kinds and proportion of different foods required to maintain the body in a normal condition. Proceeding on the same lines, we may discover the effects of an excess, actual or relative, of nitrogen; or, on the other hand, we may ascertain the results of deprivation, either partial or complete. We may also be able to see some therapeutic applications of the knowledge thus gained.

From the time of Hippocrates, and even earlier, it has been known that health and disease are largely influenced by food, and that the effects of an animal diet are different from those of a diet exclusively of vegetables. A distinction was even made between leguminous and other forms of vegetable food. It was not until our own day, however, that the practising physician possessed sufficient knowledge of the chemistry of food and of metabolism in health and disease to enable him to direct the diet of his patients upon scientific principles. Following the definition given by Hippocrates—"Medicine consists in addition and subtraction, the addition of the things which are deficient and the subtraction of those things which are redundant; he who practises this is the best physician, but he whose practice is farthest from it is the farthest removed from a knowledge of the art"—we can now prescribe viands suited to a deficiency of nitrogen in the system, or substitute others if there is an excess. To the therapeutic aspect of the subject I will now very briefly ask your attention.

Taking up the latter instance first, we find that a diet poor in nitrogen is useful in the several forms of rheumatism, in gout and lithemia, and also in recurring attacks of biliousness and bilious headache. Scurvy appears to be caused by an absolute, as well as a relative, excess of nitrogen in the food, and I have seen it caused by the use of an excessive amount of fresh meat among children in an orphan asylum. In its treatment, vegetable food relatively poor in nitrogen is usually employed. Some skin diseases, possibly of lithemic character, are only to be cured by withholding nitrogenized food. It seems possible that a liberal use of meat in the diet may have some connection with the development of cancer, a disease which appears to be on the increase, as was pointed out by Dr. R. A. Cleemann, of this Society, in his Address on Hygiene, delivered before the Medical Society of the State of Pennsylvania a few years ago. Dr. W. Mattieu Williams, in a little work on the Chemistry of Cookery, pointedly directs attention to the large consumption of meat as a cause of various forms of cancer. In families where a hereditary tendency of this kind exists, it is possible that it might be overcome by vegetarianism. Some nervous affections, notably epilepsy and chorea, are greatly benefitted by abstention from meat in the food.

Owing to the writings of Roberts, Fothergill, and others, a causative connection between a diet rich in nitrogen and some forms of kidney inflammation or degeneration is now generally recognized. And in the treatment of the various forms of Bright's disease, attention to the diet is generally admitted to be of prime importance. There is a widely spread opinion that nitrogenized food is favorable to the occurrence of inflammation, and for this there seems to be a scientific foundation. Parkes has shown that a non-nitrogenized diet causes lowered blood-pressure and diminished arterial tension. Meat, therefore, is ordinarily prohibited under the antiphlogistic treatment, as it was formerly called. In acute inflammations of mucous surfaces, especially in plethoric subjects, the use of animal food is usually forbidden. This should not be applied too strictly, however, for in some cases of subacute or chronic character, a generous and nourishing diet is necessary.

On the other hand, nitrogenized food may be prescribed where there is, from any cause, a deficiency of albuminous principles in the blood, for example in anemia or chlorosis. In phthisis, this condition is sometimes quite marked, and good results have been obtained from the "beef and hot-water" plan of treatment, and also from the use of fresh bullock's blood, or hemoglobin, which requires less digestive capacity and is more easily assimilated than muscle-tissue.

Children frequently suffer from a deficiency of nitrogen. Where an infant is reared upon condensed milk entirely, the limbs are plump, but the tissues are flabby, on account of anemia. Such children are late in getting their teeth, and have little power of resistance against disease. The addition of oat-meal, barley, or rice to the milk will often bring about marked improvement and may prevent the development of rickets. Just here I might stop to point out the fallacious character of some of the arguments based upon the comparative chemical composition of woman's milk and other foods. Leeds found in a number of specimens of woman's milk that the nitrogenous constituents varied from 4.86 to 0.85 per cent. So that one specimen of mother's milk may have six times the amount of albuminous material contained in another.[1] This shows the necessity, when the child does not thrive at the breast, of examining the milk to find out if it be deficient in nitrogenized constituents. If so, the addition of beef-meal, bovinine, or other nitrogen-containing food in an easily assimilable form is advisable.

Eczema in infants, or in sewing women, is often traceable to a deficiency of nitrogen in the food, and Dr. Rohé, of Baltimore, advises the addition of meat-broth and eggs to the diet as an essential part of the treatment. Similarly, in many syphilitic eruptions upon the skin, in broken-down subjects, good food is a necessary preliminary to any specific treatment. Neurasthenia and atonic dyspepsia, which are so often associated in the same patient, especially if he is at the same time anemic, can only be relieved by nitrogenized and fatty food, administered in a form easy of assimilation and at comparatively short intervals. On the other hand, in diabetes and in obesity the diet may be largely nitrogenous, but in this case it is because there is a desire to reduce the carbo-hydrates and not because an excess of nitrogen is particularly sought after.

To return to the children, I wish to call attention to the fact that during the period of growth and development more nitrogen is needed than after the body has assumed its full stature. Hence, school children should have a due allowance of meat, and should

1. Quoted by Starr in his "Hygiene of the Nursery," Philadelphia, 1888.

be encouraged to eat oat-meal, corn, beans, peas, and other vegetables known to contain this valuable constituent.

In the foregoing brief *résumé* of an important and interesting subject, I have not made any distinction between the nitrogenous, proximate principles of animal and vegetable origin. Chemically and physiologically they are nearly identical ; but practically there are minor differences of palatability, digestibility, and relative utility, which at present our limits will not permit us to consider.

